# Comparative Transcriptomic Analysis of Biological Process and Key Pathway in Three Cotton (*Gossypium* spp.) Species Under Drought Stress

**DOI:** 10.3390/ijms20092076

**Published:** 2019-04-27

**Authors:** Md Mosfeq-Ul Hasan, Fanglu Ma, Faisal Islam, Muhammad Sajid, Zakaria H. Prodhan, Feng Li, Hao Shen, Yadong Chen, Xuede Wang

**Affiliations:** 1 Institute of Crop Science, College of Agriculture and Biotechnology, Zijingang Campus, Zhejiang University, Hangzhou 310058, China; hasanmd12@hotmail.com (M.M.-U.H.); 21516038@zju.edu.cn (F.M.); faysal224@yaoo.com (F.I.); msajid1772@gmail.com (M.S.); rajugenetics2003@gmail.com (Z.H.P.); seawind-1@zju.edu.cn (F.L.); shenhao1721@163.com (H.S.); sdaucyd@163.com (Y.C.); 2 Examination Controller Section, Hajee Mohammad Danesh Science and Technology University, Dinajpur 5200, Bangladesh

**Keywords:** drought, transcriptome, cotton, stress physiology, mechanism

## Abstract

Drought is one of the most important abiotic stresses that seriously affects cotton growth, development, and production worldwide. However, the molecular mechanism, key pathway, and responsible genes for drought tolerance incotton have not been stated clearly. In this research, high-throughput next generation sequencing technique was utilized to investigate gene expression profiles of three cotton species (*Gossypium hirsutum*, *Gossypium arboreum*, and *Gossypium barbadense* L.) under drought stress. A total of 6968 differentially expressed genes (DEGs) were identified, where 2053, 742, and 4173 genes were tested as statistically significant; 648, 320, and 1998 genes were up-regulated, and 1405, 422, and 2175 were down-regulated in TM-1, Zhongmian-16, and Pima4-S, respectively. Total DEGs were annotated and classified into functional groups under gene ontology analysis. The biological process was present only in tolerant species(TM-1), indicating drought tolerance condition. The Kyoto encyclopedia of genes and genomes showed the involvement of plant hormone signal transduction and metabolic pathways enrichment under drought stress. Several transcription factors associated with ethylene-responsive genes (*ICE1*, *MYB44*, *FAMA*, etc.) were identified as playing key roles in acclimatizing to drought stress. Drought also caused significant changes in the expression of certain functional genes linked to abscisic acid (ABA) responses (*NCED*, *PYL*, *PP2C*, and *SRK2E*), reactive oxygen species (ROS) related in small heat shock protein and 18.1 kDa I heat shock protein, *YLS3*, and *ODORANT1* genes. These results will provide deeper insights into the molecular mechanisms of drought stress adaptation in cotton.

## 1. Introduction

Drought is one of the major abiotic stresses that greatly affects plant growth and crop production worldwide. It also inhibits metabolism, development, and growth of many crops [1,2]. Under drought tolerance, plants maintain turgor and persist metabolism in cells even at low water potential, mainly by synthesis of osmolytes or/and compatible solutes [3]. Production of different compatible osmolytes such as proline, glycine-betaine, trehalose, as well as antioxidant enzymes like superoxide dismutase (SOD), catalase (CAT), and ascorbate peroxidase (APX) are proven to protect plant cells from immediate cellular damage by regulating gene expression level [4]. It is essential to analyze the function of drought stress-inducible genes enriched in drought related pathways, but there is also a need to understand the expression of genes that are related to drought stress avoidance. Thousands of genes and many signaling pathways have been identified in plants during drought stress [2,5,6]. Under drought avoidance and tolerance mechanisms, plants maintain high water status during stress by reducing transpirationand/or improving water absorption, sustaining turgor, and maintaining a steady metabolism rate even at low water potential [7].

Cotton (*Gossypium* spp.) is considered as a cash crop, planted widely in China. As extra economic value is captured from cotton seed and its related cotton byproducts, cotton lint represents 90% of cotton’s total economic value [8]. China, the United States (US), and India provide most of the world’s cotton; more than 15.95 million metric tons of cotton as lint and 29.26 million metric tons of cotton as seeds were exported in 2014 (FAOSTAT, www.faostat.fao.org). Cotton plants frequently encounter unfavorable growth conditions, i.e., drought, salinity, and heat stress. Cotton is a semi-arid to subtropical crop whose aerial parts have changed mechanisms conferring moderate tolerance to drought stress [9,10,11]. The cotton plants adjust changeable soil moisture levels by its extensive root system. All over the world, plant breeding for drought tolerance in cotton has resulted in a broad range of adapted genotypes [11,12,13].

The molecular mechanism of drought tolerance and resistance has been studied at extensive levels in cotton. Drought stress effects on plants aerial parts such as leaf stem and flowering tissues [9,10,11], furthermore, plant hormones such as abscisic acid (ABA), auxin, cytokinins, and gibberellin play a key role in plant adaptation to drought stress [1,6,14,15]. The cotton plant accumulates a high concentration of ABA under drought stress condition which modulates the expression of a number of drought-related genes. Drought-related gene expressions mechanisms lead to the reprogramming of different physiological, biological, and metabolic processes in agreement with cotton plants. Previously, many researchers used model plants such as *Arabidopsis* and rice to identify a large number of genes that are concerned in metabolism, signal transduction, osmo-regulation, and stress-responsive gene regulation [8,16,17]. Recently, some researchers have determined drought stress-related gene expressions on cotton using a range of technical approaches [18,19].

Drought stress tolerance is a multi-genetic attribute connected with morphophysiological and molecular characters, as well as the genetic improvement of crops on the basis of selection processes [20]. Previously, researchers have improved the drought tolerance in cotton species through conventional breeding programs. Cotton production performs well under drought stress on the basis of selection methods, and this selection normally categorizes some traits for screening to drought tolerance [15,21]. Drought tolerance in cotton depends on some characteristics such as anatomical, physiological, biochemical, and molecular parameters, but this selection procedure is more laborious and time-consuming. Few breeders recommend a vast approach for the improvement of breeding efficiency towards drought stress [22]. Previously, some researchers observed that over-expression of *GhZFP1*, *GhMPK2*, and *GhMKK5* in tobacco affected the transgenic plant salinity and drought stress tolerance cotton (*GhCIPK6)*, as well as maize (*ZmCIPK5*) [23,24,25]. High throughput sequencing technology has also been used to search for drought-responsive genes in cotton. Furthermore, the improved sequencing technology and achievement of diploid cotton genome sequencing has made this investigation much easier [10,26,27]. Nowadays, rapid progresses in RNA-Seq and connected bioinformatics tools have afforded revolutionary tools for transcriptomic research on plant genomes [28,29]. The transcriptomic profiles of drought responses have been surveyed in *Ammopiptanthus mongolicus* [30] using RNA-seq. Several researchers detected differentially expressed genes from drought stressed root and leaf tissues in tetraploid cotton using cDNA-AFLP [8]. In our previous study [21], we found the drought resistant ability in different cotton genotypes, and observed that the drought tolerance is due to the laidback work of several morpho-phyosio biochemical and molecular mechanisms. In the present study, we analyzed a genome-wide comparative analysis of contrasting cotton species to identify drought stress-responsive genes and biological pathways that might advance our understanding of tolerance mechanisms in drought-stressed cotton plants. The relative expression profiles of three cotton species with differential drought stress tolerance will be useful in consolidating our knowledge of the molecular mechanisms of cotton in response to drought stress.

## 2. Results

### 2.1. Transcriptome Sequencing, Data Statistics and Evaluation, and Reads Mapping

Drought-responsive molecular mechanisms were examined in three cotton species (TM-1, Zhongmian-16, and Pima4-S) to identify potential candidate genes involved in drought tolerance, through deep RNA sequencing of *Gossypium spp* seedlings subjected to drought was performed using Illumina sequencing platform. In total, 6 libraries were constructed and sequenced using the Hisat2 (v2.0.1) sequencing platform and 3.26 × 10^8^ raw reads were generated. After removing the linker and low-quality data, we obtained 3.22 × 10^8^ (99%) clean reads and an average of 5.36 × 10^7^ clean reads. We also obtained an average 44.36 × 10^7^ total mapped reads, 10.75% multiple mapped reads, and 71% uniquely mapped reads. Phred mass fraction Q20 and Q30 (error rate 1% and 0.1%, respectively) ranged from 96.26 to 96.76% and 91.18 to 92.17%, with an average GC content of 42.99%. We aligned 81% of the valid data to the reference genome via software Hisat2 (v2.0.1) (Table 1). The distribution of reads among exonic and intergenic regions of three cotton genomes, RPKM density distribution and RPKM box diagram revealed that almost 90% of the exonic region suggested that the density of the detected genes followed a standard normal distribution percent error rate saturation curve (Figures S1–S4). These results indicated high quality and logical reproducibility of our sequencing data.

### 2.2. Study of Gene Expression in Three Cotton Species Under Drought Stress

**Figure 1:**
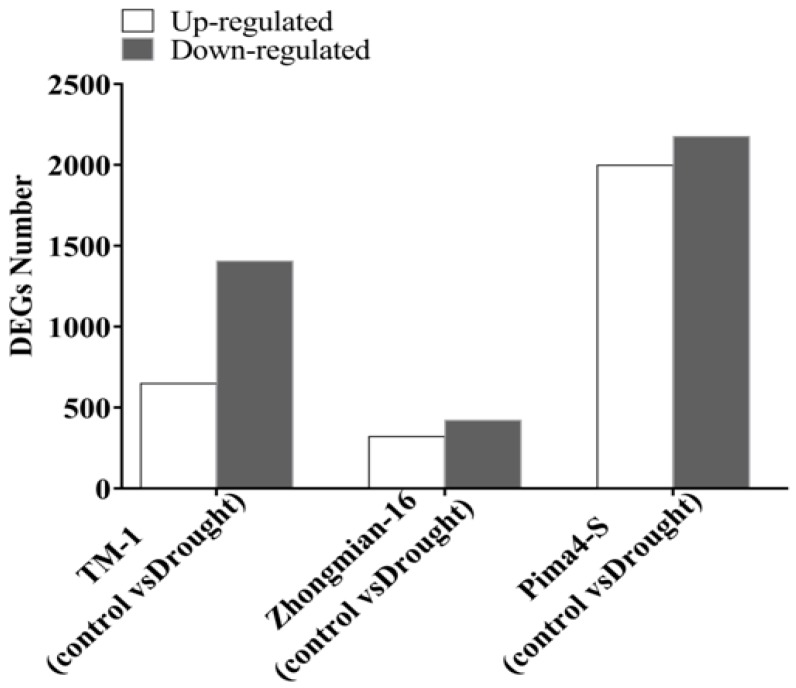
Differential gene expression (DEGs) in three cotton species.

**Figure 2:**
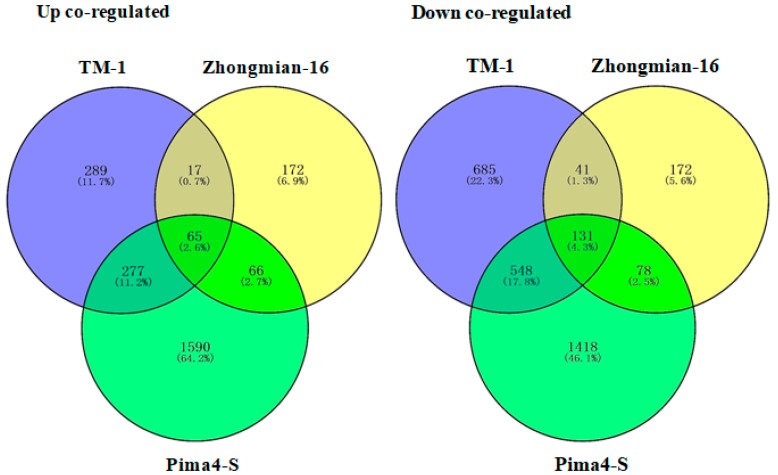
Venn diagram of 6968 unigenes, identified as differentially expressed in the three cotton species. The number of DEGs in each comparison shows in each circle; the number of up co-regulated (65 DEGs) and down co-regulated (131 DEGs) present in each comparison.

**Figure 3:**
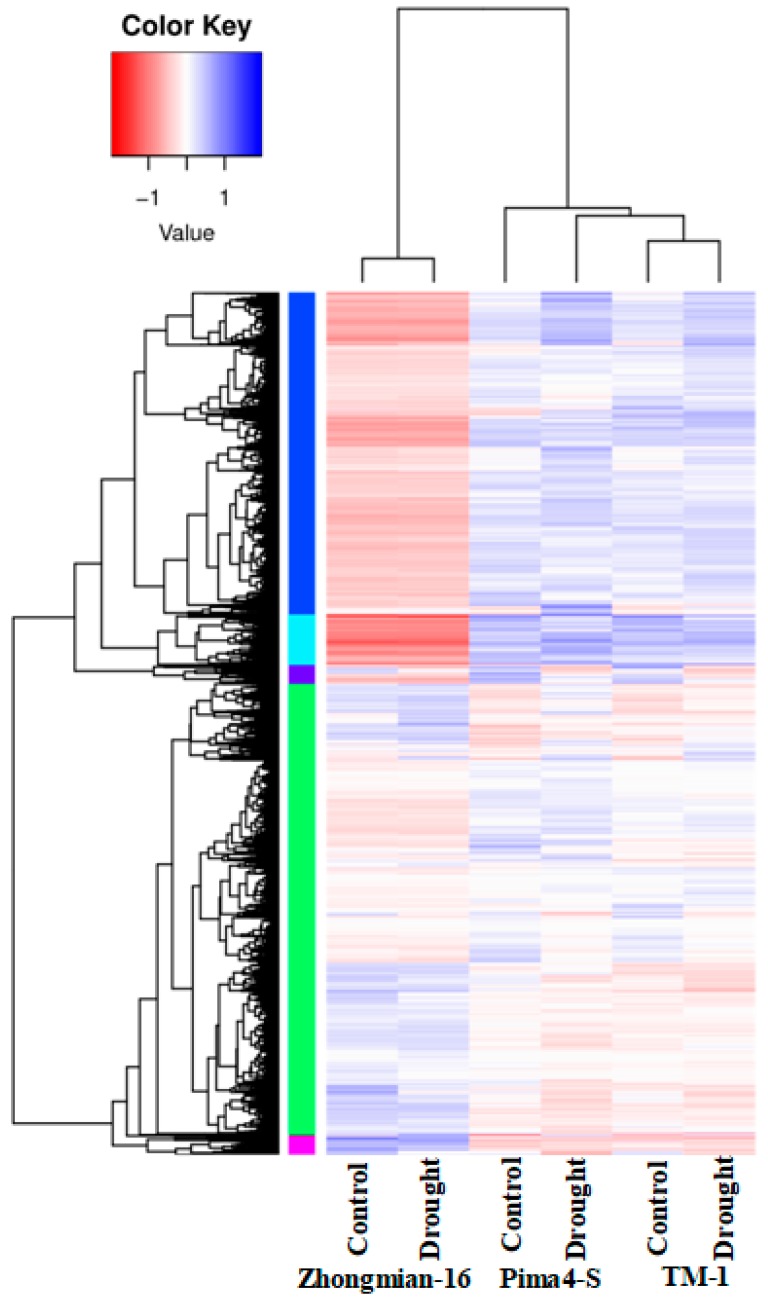
Transcript levels of differentially expressed genes (DEGs) in three cotton species under drought stress condition in heat maps. Heat map of all DEGs, columns, and rows in heat maps represent samples and DEGs, respectively. Blue color indicates up-regulated and red color indicates down-regulated (*p* < 0.05).

To understand differential drought tolerance across the three cotton species, differential expression analyses were performed on samples collected from TM-1 control and drought, Zhongmian-16 control and drought, and Pima4-S control and drought. In total, 6968 differentially expressed genes (DEGs) expressed (*q* ≤ 0.05) between three drought treatments and the control samples were obtained (Table S1). Of these, DEGs were expressed between the TM-1 control and drought 2053, Zhongmian-16 control and drought 742, and Pima4-S control and drought 4173 (Figure 1). Among the DEGs, 648, 320, and 1998 were found to be up-regulated (*q* ≤ 0.05) and 1405, 422, and 2175 were found to be down-regulated (*q* ≤ 0.05) in the TM-1 control and drought, Zhongmian-16 control and drought, and Pima4-S control and drought, respectively (Figure 1, Figure 2 and Figure 3). Venn diagram shows that a total of 6968unigenes were identified as differentially expressed in the three cotton species, TM-1, Zhongmian-16, and Pima4-S (Control-vs-Drought) (Figure 2). The transcript levels of DEGs in three cotton species under drought stress condition are shown in heat maps (Figure 3). We were also compared co-up regulated and co-down regulated DEGs in TM-1, Zhongmian-16, and Pima4-S under drought-stress condition (Figure S5). The distribution of DEGs on MA plot and volcano plots are presented in Figure S6.

### 2.3. Gene Ontology (GO) Enrichment Analysis

**Figure 4:**
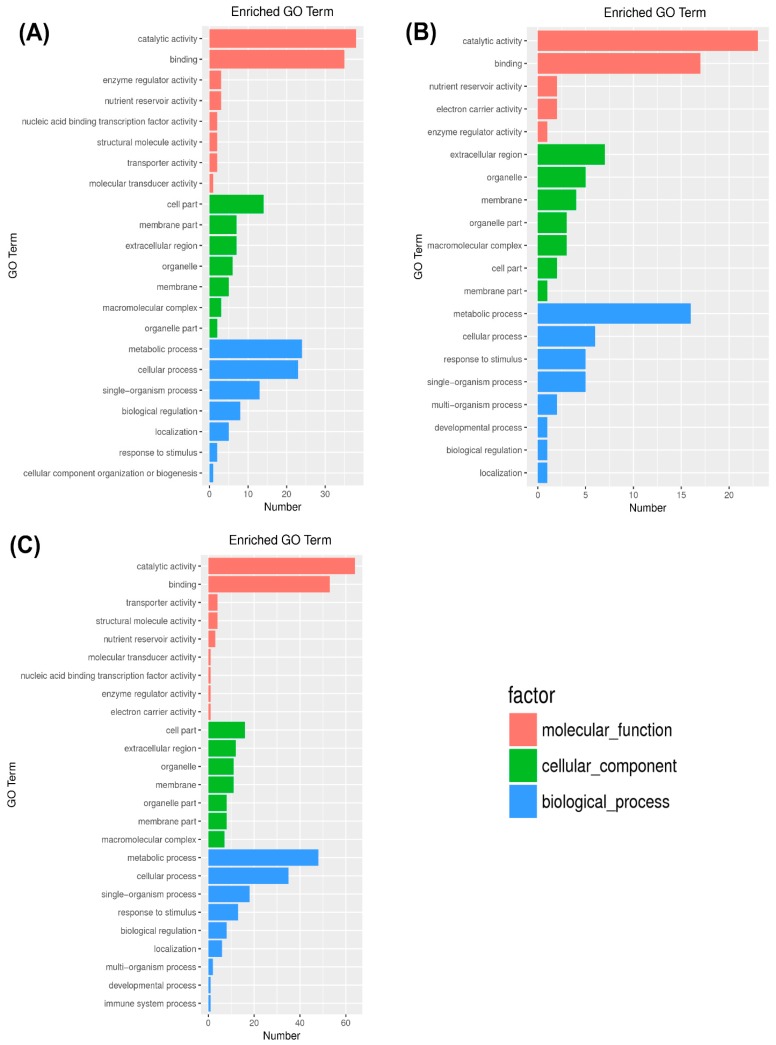
Gene ontology enrichment in all DEGs. Here, (**A**) TM-1 control and drought, (**B**) Zhongmian-16 control and drought, and (**C**) Pima4-S control and drought.

Gene ontology (GO) is a worldwide reliable gene functional classification system. For the functional characterization of DEGs (TM-1, Zhongmian-16, and Pima4-S) in control and drought stress treatments, GO terms include mainly cellular components, molecular functions, and biological processes. A sum of 6968 transcripts were annotated and classified into 22, 20, and 25 functional groups in TM-1, Zhongmian-16, and Pima4-S, respectively (Figure 4). TM-1 includes 8, 7, and 7 DEGs in molecular functions, cellular components, and biological processes, respectively. In Zhongmian-16 and Pima4-S, together with 5, 7, 8 DEGs and 9, 7, 9 DEGs were expressed in molecular functions, cellular components, and biological processes, respectively (Table S3). In TM-1, under the cellular component, the GO category “cell” expressed 18 DEGs (62.1% out of 29; GO:0005623), “cell part” expressed 18 DEGs (62.1% out of 29; GO:0044464), “extra cellular region” expressed 7 DEGs (24.1% out of 29; GO:0005576), “apoplast” expressed 6 DEGs (20.7% out of 29; GO:0048046), molecular function “lyase activity” expressed 8 DEGs (13.3% out of 60), and biological process “photosynthesis” expressed 4 DEGs (8.0% out of 50). Under the cellular component, “extra cellular region” contained 7 DEGs (58.3% out of 12; GO:0005576), “apoplast” contained 5 DEGs (41.7% out of 12; GO:0048046), “chloroplast envelope” contained 2 DEGs (16.7% out of 12; GO:0009941), and biological process “oxidation-reduction process” contained 9 DEGs (33.3% out of 27; GO:0055114), “carbon utilization” contained 2 DEGs (7.4% out of 27; GO:0015976), and “response to cold” category contained 2 DEGs (7.4% out of 27; GO:0009409) in Zhongmian-16 under drought-stress conditions. In Pima4-S under the cellular component, category “cell” contained 25 DEGs (58.1% out of 25; GO:0005623), “cell part” contained 25 DEGs (58.1% out of 29; GO:0044464), “extra cellular region” contained 12 DEGs (27.9% out of 43; GO:0005576), “apoplast” contained 7 DEGs (16.3% out of 43), and “lyase activity” contained 12 DEGs (12.8% out of 94; GO:0016829) under molecular functions. Interestingly, Zhongmian-16 and Pima4-S molecular function and biological process were absent (Table S3). The present study implies that the biological process might specifically be involved in contributing to drought tolerance in the TM-1 cotton species.

Enriched (*q* ≤ 0.05) GO terms were observed in the foundation categories of metabolism, oxidation-reduction, and photosynthesis, and these terms were significantly over- or under-represented in different cotton species under droughtstress treatment. These results suggest that the expression of proteins associated with these GO terms was strongly affected by drought. Enriched GO term TM-1 (control and drought), Zhongmian-16 (control and drought), and Pima4-S (control and drought) species in the molecular function class belonged to “catalytic activity” and the second was “binding” (Figure 4). In the oxidation-reduction category, the following GO terms were significantly over-symbolized: the biological processes of oxidation-reduction and the molecular functions of oxido-reductase activity, including acting on peroxide as an acceptor, acting on the acting on paired donors, acting on single donors with the incorporation of molecular oxygen, and the incorporation or reduction of molecular oxygen (Table S2). These findings indicated that oxidation-reduction reactions and oxidoreductase activity were enhanced. In the categories related to photosynthesis, the down-regulated GO terms included the biological process photosynthesis and the cellular components of photosystem, photosystem I, photosystem I reaction center, photosystem II, and photosystem II oxygen-evolving complex, indicating that photosynthetic functions were inhibited. GO terms for biological processes that occurred in response to stress and negative regulation of catalytic activity and molecular function were highly enriched.

In the molecular function category, a significant proportion of the clusters were classified as binding, catalytic activity, with transcription factor activity and sequence specific DNA binding. In the biological process category, the major subcategories were metabolic process, cellular process, and single organism process. In the cellular component subcategory, the largest proportion sub-categories consisted of the membrane cell with cell part. Moreover, GO enrichment functions of all DEGs (up- and down-regulated) were analyzed through the results which showed that a significant number of genes were involved in binding and catalytic activity followed by single organism process along with single organism metabolic process (Table S4, Figure S7, and Figure 4).

### 2.4. Kyoto Encyclopedia of Genes and Genomes and DEGs

For further investigation, the mechanisms involved in drought stress of the TM-1 tolerant species, the unigenes orientations of metabolic pathways, were studied by searching the KEGG database through bar plot analysis. In total, 13,624 genes were assigned and among them only 483, 189, and 964 genes were expressed in TM-1, Zhongmian-16, and Pima4-S, respectively. In TM-1, expressed genes were found to assign to five definite pathways. They were included in cellular processes, environmental information processing, genetic information processing, and metabolism and organismal systems (Figure 4). Among these pathways, ko04075 (plant hormone signal transduction), ko00500 (starch and sucrose metabolism), ko00260 (glycine, serine, and threonine metabolism), and ko00250 (alanine, aspartate, and glutamate metabolism) were enriched among the up-regulated DEGs, whereas photosynthesis was enriched among the down-regulated DEGs. These results indicate that drought stress induced the signal transduction of plant hormones, which had strong effects on biosynthesis and metabolism and led to a decline in photosynthesis. In addition, certain pathways enriched by DEGs occurred under the drought stress treatment (Table S5). 

**Figure 5:**
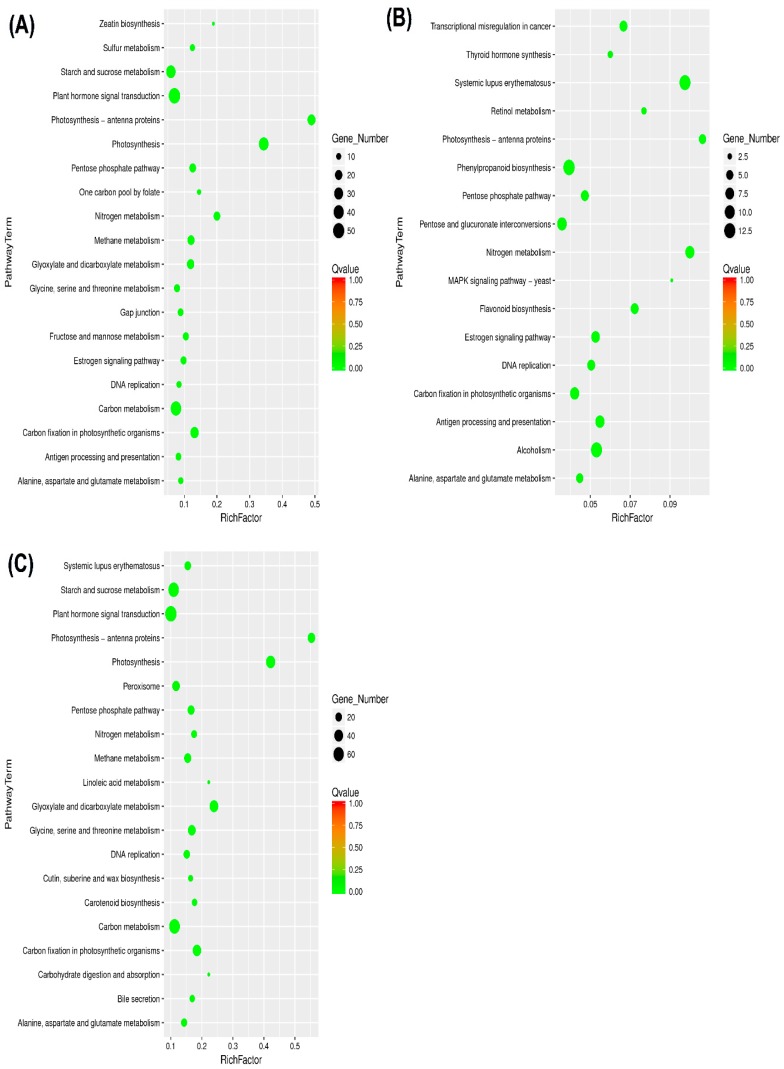
Scatter plot of KEGG pathway enrichment statistics. Here, (**A**) TM-1 control and drought, (**B**) Zhongmian-16 control and drought, and (**C**) Pima4-S control and drought. Rich Factor is the ratio of differentially expressed gene numbers annotated in this pathway term to all gene numbers annotated in this pathway term. Greater Rich Factor means larger demanding. Q-value is corrected *p*-value ranging from 0~1, and its lesser value means greater intensiveness. We have only displayed the top 20 pathway terms enriched by the KEGG database.

We also analyzed the scatter plot of significant KEGG enrichment function (Figure 5). This analysis represented the function of the Rich Factor (the number of differential genes annotated to the function divided by the number of genes annotated to the function) and the closer to red, the more the differential genes contained under each function were represented by the size of the dots. Here, we selected only the top 20 KEGGs with the significantly highest level of enrichment. Among them, DEGs were significantly enriched in important KEGG pathways related to drought stress tolerance, such as ko04075 (plant hormone signal transduction), ko00260 (glycine, serine, and threonine metabolism), ko00250 (Alanine, aspartate and glutamate metabolism), ko00500 (starch and sucrose metabolism), etc.

### 2.5. Identification of Drought-Responsive Genes and Transcriptional Factors (TFs)

GO enrichment and KEGG pathway analysis indicated that these stress-induced DEGs in TM-1 were widely involved in plant hormone signal transduction, carbon metabolism, starch and sucrose metabolism, glyoxylate and dicarboxylate metabolism, pentose phosphate pathway, glycine, serine and threonine metabolism, fructose and mannose metabolism, alanine, aspartate and glutamate metabolism photosynthesis, and other biological processes (Table S3). In TM-1, plant hormonal signal transduction pathway involved 51 DEGs (10.56%) (21 up-regulated and 30 down-regulated). Under GO-enrichment, we identified 4 genes in biological process subgroup photosynthesis. Transcription factors (TFs) appear to have a major effect on drought-responsive genes; one of the objectives of this study was to identify drought-inducible TFs (Table S2).

TFs were differentially expressed (up-regulated) in TM-1 cotton species in response to drought stress, including ethylene-responsive transcription factor *CRF1*, transcription factor *bHLH55*, *bHLH87*, *MYB44*, *DOF5.7*, *MUTE*, *FAMA*, *bZIP53* zinc finger CCCH domain-containing protein 66, *NAC, ZAT5*, *AZF1*, all are up-regulated in TM-1 drought tolerant cotton species only. A total of 36 DEGs in TM-1 were identified to be involved in drought tolerance. Among them, DEGs were involved in oxidative stress, binding, biosynthesis, catalysis, development, drought stress tolerance response, hydrolase activity, inhibitor, kinase, and oxidoreductase. These genes were identified in TM-1 tolerant cotton species.

### 2.6. Justification of DEG-Based Gene Expression

**Figure 6:**
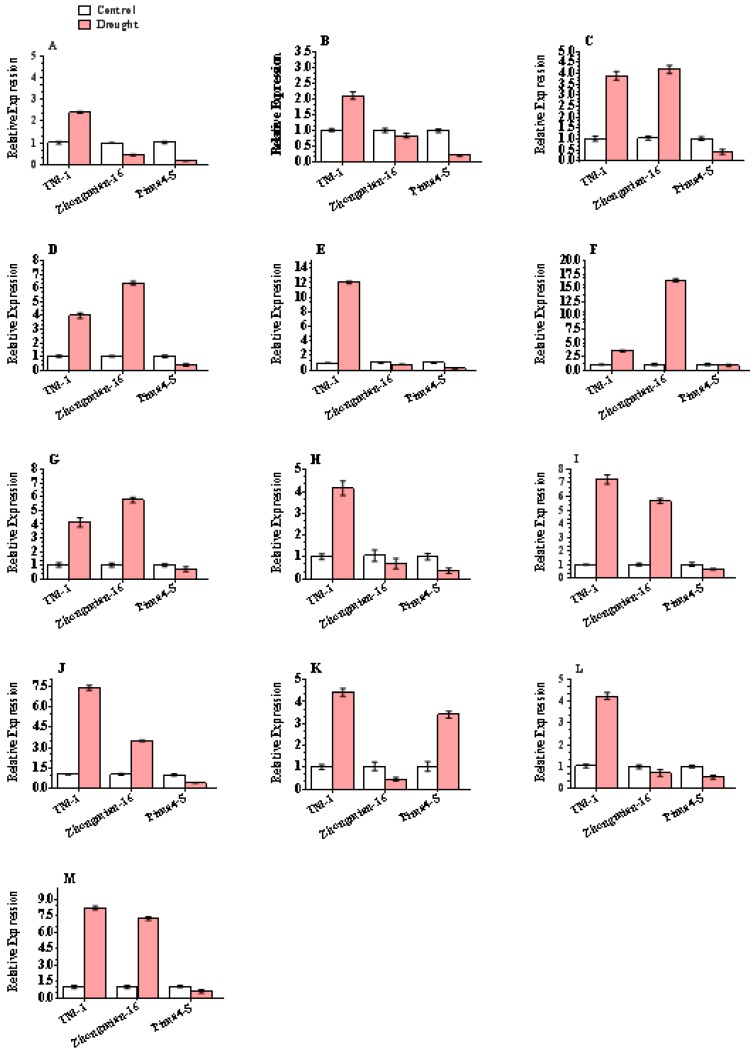
Relative abundance of selected transcripts as determined by qPCR. Expression profiling of selected 13 DEGs were performed using quantitative real time PCR. Here, (**A**) indole-3-acetic acid-amidosynthetase GH3.17, (**B**) ribulose bisphosphate carboxylase/oxygenase activase 2% 2C chloroplastic, (**C**) transcription factor *HEC2*, (**D**) glucan endo-1% 2C 3-beta-glucosidase 5, (**E**) inositol oxygenase 1, (**F**) probable trehalose-phosphate phosphatase D, (**G**) protein DETOXIFICATION 49, (**H**) 21 kDa, (**I**) pectinesterase, (**J**) arginine decarboxylase, (**K**) leucine-rich repeat receptor protein kinase *PXC2*, (**L**) secoisolariciresinol dehydrogenase, and (**M**) thaumatin protein genes in the leaves of three cotton species, TM-1, Zhongmian-16, and Pima4-S, are presented. The relative abundance (Y-axis) was calculated using the 2^−ΔΔ*C*t^ method. Three cotton species seedlings were subjected to drought stress. The mean of three independent biological replicates is presented.

To verify the reliability of the drought-responsive gene expression profiles for DEGs, 13 genes were confirmed by quantitative real-time PCR using gene-specific primers (Table S5). These selected genes encoded indole-3-acetic acid-amidosynthetase GH3.17, ribulose bisphosphate carboxylase/oxygenase activase 2% 2 C chloroplastic, transcription factor *HEC2*, glucan endo-1% 2C 3-beta-glucosidase 5, inositol oxygenase 1, probable trehalose-phosphate phosphatase D, protein *DETOXIFICATION 49*, 21 kDa, pectinesterase, arginine decarboxylase, leucine-rich repeat receptor protein kinase *PXC2*, secoisolariciresinol dehydrogenase, and thaumatin protein genes in the leaves of three cotton species, TM-1, Zhongmian-16, and Pima4-S, are presented. It is obvious that the expression patterns of the selected drought-responsive genes produced by quantitative real-time PCR were consistent with RNA-seq data (Figure 6). We determined the comparative gene expression by the relative threshold cycle (Ct) method (2^−ΔΔCt^ method).

## 3. Discussion

Drought is one of the most vital abiotic stresses that limits crop growth and agricultural productivity [31]. Drought stress significantly affects morpho-physiological, biochemical, and cellular damage, along with disturbances in nutrient uptake drought stressthat also affected photosynthetic apparatus in sunflower varieties [32]. Drought also influences osmotic stress, oxidative damage, and stomatal closure [33,34]. In our previous study, we identified that TM-1 had the highest tolerance to drought stress on the basis of plant growth, chlorophyll content, net photosynthetic rate (*P*n), stomatal conductance (*G*s), maximum photochemical efficiency PSII (*F*v/*F*m), and relative water content compared to Zhongmian-16 and Pima4-S [21]. We also investigated biochemical properties (proline, soluble proteins, and soluble sugars content), and antioxidant activity superoxide dismutase (SOD), peroxidase (POD), ascorbate reductase (APX), and catalase (CAT) significantly increased in the drought-tolerant species (TM-1) [21]. The relative expression level of some selected genes increased to higher levels in TM-1 than in Zhongmian-16 and Pima4-S under drought stress conditions [21]. It is important to explore the functions of stress-responsive genes and to understand the molecular mechanisms responsible for stress tolerance. In the current study, we have provided information about the exploration of drought resistance mechanisms in cotton at transcriptomic levels. RNA-Seq analysis is the most effective measurement of transcript profiling and annotation as well as identification of genes in many plant species [28]. This study also aims to figure out differences in transcription/translation levels in cotton leaves to identify drought-responsive genes and biological pathways, as well as to assess the drought stress responseof three cotton species, i.e., TM-1, Zhongmian-16, and Pima4-S using RNA-seq analyses.

### 3.1. Drought-Related Genes in DEGs

Drought-responsive genes or proteins that instantly protect the cell are mostly associated with metabolic pathways; they act as regulators that control stress signaling, but also forming a set of elements responding to abiotic stress [35,36]. Cell wall associated kinase and *CAMTA*/*CAMK* are vital in plant responses to abiotic stress [37,38]. Protein phosphatase 2C (*PP2C*) belongs to a group of phosphatases involved as a negative regulator of ABA signaling [39]. In our study, different DEGs were identified from three drought induced cotton species, 10 genes encoding *CAMTA*/*CAMK* kinases were regulated, and five genes encoding WAK receptor kinases were drought related. Among them, two genes were up-regulated in TM-1 and three genes down-regulated in Pima4-S. Nine protein phosphatase 2C (*PP2C*) genes were up- and three down-regulated in TM-1 eight genes were up- and down-regulated in Pima4-S cotton species (Table S1). Aux/IAA and GA are phytohormones playing a vital role in normal plant growth and development, and in reacting to abiotic stresses [40,41]. Our study detected in TM-1 (one up- and two down-regulated), Zhongmian-16 (4 up-regulated), and Pima4-S (two up- and two down-regulated) gene encoding Aux/IAA and GA-encoding genes in TM-1 (one up- and five down-regulated), Zhongmian-16 (two up- and one down-regulated), and Pima4-S (three up- and five down-regulated) (Table S1). Many defensive proteins have a vital role in protecting plants from injury caused by drought stress. For example, late embryogenesis abundant proteins (LEAs), which reduce water loss in plant cells and increase their WUE under stressful environments [8]. Genes encoding protective proteins that were up-regulated in all three species in our study included fifteen coding for LEAs and universal stress proteins (USP) in TM-1 (one up- and two down-regulated) and Pima4-S (five up- and 2 down-regulated) under drought conditions (Table S1). In cotton, 71 members of aquaporin proteins were identified which were responsible for cellular water uptake and other small molecules transportation across cell membranes [8,42].

The transporter proteins and ion channels also play an important role in drought tolerance [43]. Here, we detected fourteen genes encoding aquaporins, various transporters, ion channels, and sugar transporter in all three cotton species under drought stress condition (Table S1). The ATP-binding cassette transporters (ABC) members are involved in abiotic stress and functions under abiotic stresses. Most of them are transporting terpenoids and their imitative, as reported in *Arabidopsis* and other plants [44,45]. In our study, we detected four ABC family transporters, sugar transporter (*ERD6*) up-regulated in tolerant species TM-1 under drought stress (Table S1). The expressions of expansin genes were found to be up-regulated in TM-1 under drought stress (Table S1). It was reported that expansin protein activity increased in the plants were induced by drought stress [46]. The higher expression of expansin proteins under drought stress changes the cell wall structure or chemistry of tolerant genotypes that may assist turgor pressure [47]. Glutamate dehydrogenase was enriched in TM-1 under drought stress. Osmo-protectant includes one such as proline, which contributes to osmotic adjustment and thereby improved drought stress tolerance in plants [48]. A larger increased relative abundance of enzymes such as Galactinol synthase and galactinol sucrose galactosyl transferase involved in galactinol (osmolyte) biosynthesis was observed only in tolerant species TM-1. TF families, including *MYB*, *NAC*, *WRKY*, *bHLH*, *bZIP*, *ERF*, zing finger CCCH domain containing protein, zing finger Dof, and ethelyne-responsive transcription factor (*RAP*, *CRF1*) were differentially expressed in our three examined cotton species under drought stress (Table S1). These TFs may play crucial roles in biotic and abiotic stress responses and our results were in line with previous studies, where *bZIP*, *AP2/EREBP*, *MYB*, *NAC*, *WRKY*, and zinc-finger protein TFs had played crucial roles in the plant response to drought stress [49,50]. The antioxidant defense system in plants under drought stress is composed of reactive oxygen species (ROS) scavenging enzymes. Among them, CAT, SOD, and APX are essential antioxidant elements to remove ROS and act synergistically to work against oxidative damage caused by drought stress [51]. All DEGs identified in drought-treated TM-1, Zhongmian-16, and Pima4-S were involved in drought stress. The transcription of many of them was regulated, suggesting that these genes may play a vital regulatory function in drought response.

### 3.2. Gene Ontology

GO enrichment analysis revealed that the DEGs were enriched in response to various stresses, such as drought stress, osmotic stress, salt stress, other organisms, oxidative stress, starvation, water deprivation, and wounding. It is well known that drought stress is a typical abiotic stress [52]. Osmotic adjustment is a vital physiological mechanism for adaptation to drought [53], which helps to repair cellular apparatus to alleviate plant cell damage and enhance stress resistant. Moreover, drought stress affects plant growth and development generally through photosynthetic decline, osmotic hindrance, and interfere with nutrient availability [54]. It has been reported that drought, salt, heat, cold, and oxidative stress may be convoy by the formation of ROS (superoxide, hydrogen peroxide, and hydroxyl radicals), thus causing cellular damage and inhibition of photosynthesis [55]. Enriched (*q* ≤ 0.05) GO terms were observed in the foundation categories of metabolism, oxidation-reduction, and photosynthesis, and these terms were significantly over or under represented in different cotton species under drought stress treatment. In our study, we observed that three cotton species have a commonly cellular component category at present. Interestingly, in Zhongmian-16 and Pima4-S the molecular function and biological process categories were absent, respectively (Table S3). Under biological process subcategory photosynthesis, there present four DEGs (*LOC_107959556*, *LOC_107908551*, *LOC_107954794*, and *LOC_107958776*) in tolerant species, TM-1. The present study suggests that the biological process might specifically be involved in drought tolerance in TM-1 cotton species.

### 3.3. KEGG Pathway Involved in Drought Stress

**Figure 7:**
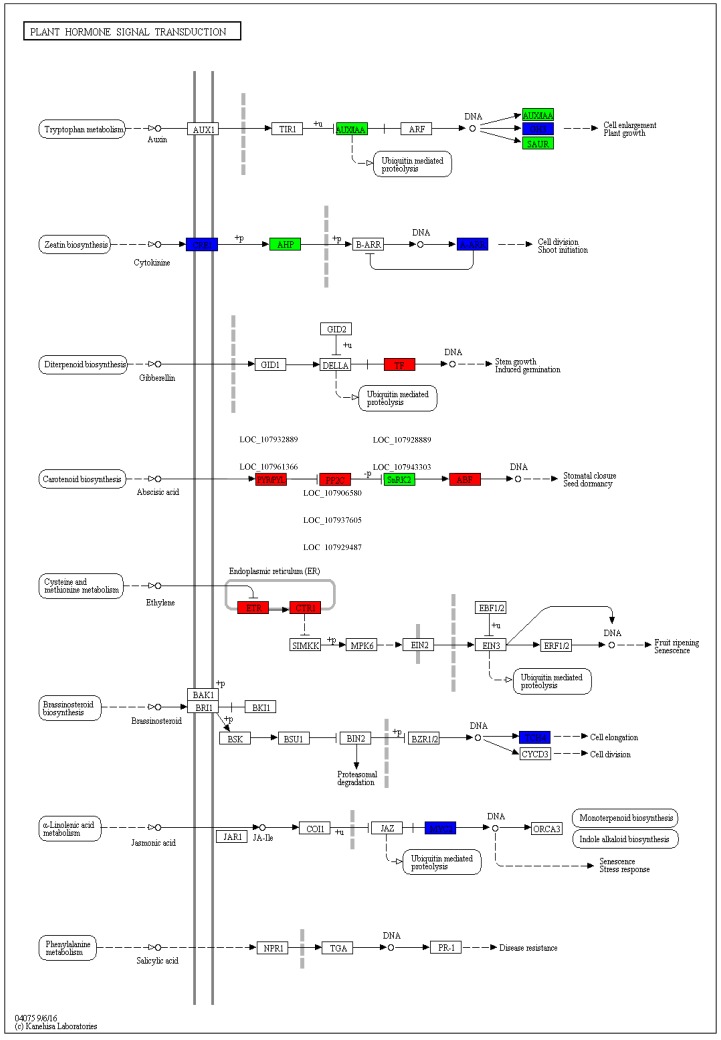
KEGG pathway image of plant hormonal signal transduction DEGs. KEGG search and color pathway analysis of significant differentially expressed transcripts in plant hormonal signal transduction pathway in TM-1 cotton species leaf under drought stress. Enzymes coded red are up-regulated and blue are down-regulated, and purple genes denote the reference pathway.

Plant hormones are important signal molecules that are produced at very low concentrations produced in plants, but involved in a wide range of processes, including the formation of flowers, stems, leaves, ripening of fruits, and responding to drought stress [43]. In this research, many genes of hormonal signaling pathways, including auxin, serine/threonine protein kinase, abscisic acid; and ethylene were found to be involved in the drought response in cotton (Figure 7, Table S4). Phytohormones like ABA play an important role in plant adaptation to abiotic stresses, such as drought [56]. Although, ABA accumulates in plant cells under drought stress and endorses stomatal closure, as well as the expression of drought-responsive genes to be strongly related to drought tolerance mechanisms in plants [57]. Many researchers have also reported that the gene encoding *NCED3* (9-cis-epoxycarotenoid dioxygenase) is a vital enzyme for ABA production and is over-expressed in plants under drought stress [58,59]. In our present study, we observed that *NCED3* (9-cis-epoxycarotenoid dioxygenase) was up-regulated in TM-1 and down-regulated in Pima4-S species under drought stress. Quan et al. [60] reported that ABAs are important phytohormones which have a drought stress response in Masson pine, when the drought stress increases ABA content also increases. Accordingly, a key gene linked to ABA and increasing ABA was found to be up-regulated under drought stress in cotton as well as being advantageous to the development of plant drought stress tolerance. Furthermore, three major components connected with hormonal signal transduction were also found to be differentially expressed in our current study. *PYL* (*LOC_107932889* and *LOC_107961366*) is a gene encoding ABA receptor that takes part in trigger ABA responses [61] and was up-regulated only in the tolerant species TM-1 under drought stress condition. However, a gene encoding PP2C, type 2C protein phosphatase (*LOC_107906580*, *LOC_107937605* and *LOC_107929487*), a negative regulator that inactivates *SrK2E*/*SnRK2*/*OST1* protein kinases [62], was obviously repressed under drought stress in TM-1 species. In addition, two genes encoding *SrK2E*/*SnRK2*/*OST1* (*LOC_107928889* and *LOC_107943303*), which improves drought tolerance by enhancing ABA signaling [63], were activated under drought stress in TM-1. The ABA signaling pathway involves many important genes related to ABA response and its double-regulatory system endorsing the interface between *PYL* and *PP2C*, thus leading to *SnRK2* activation and *PP2C* inhibition in cotton. These results indicated that many important genes related to ABA, all of which are involved in ABA signaling pathway and its double-negative regulatory system, promote the interface among *PYL* and *PP2C*, thereby leading to *PP2C* embarrassment and *SnRK2* activation in cotton. These findings are reliable with those of previous studies in which drought stress tolerance was putatively connected with ABA signaling networks in plants [64]. The ABA-mediated response pathway was markedly activated under drought stress in tolerant species TM-1; thus, phytohormone ABA plays a central role in drought stress responses in cotton (TM-1).

### 3.4. Drought-Responsive Genes and TFs Present in TM-1 Species

Aquaporins are membrane channel proteins that facilitate water transport across cell membranes. We observed aquaporin *PIP1; 2* proteins were up-regulated in tolerant species (TM-1). It was reported that *PIP1; 2* was driven through two various promoters, conferring improved tolerance to drought stress [42,65]. Serine/Threonine Protein Kinase *CTR1* is one of the major *MAPKKK* that have been identified to respond to cold and drought [66]. *MAPKs* have been isolated from different plant species that are concerned not only in many signal transduction processes but also cellular processes including development, hormonal, and abiotic signaling [67]. Bleecker and Kende [46] reported that expansin protein activity increased in the plants under drought stress. The higher expression of expansions when induced drought stress changes the cell wall structure of the tolerant genotype perhaps assists turgidity [47]. We identified *CTR1* (3 gene), expansin-1 up-regulated in TM-1 tolerant species under drought condition. The *CBLs/CIPKs* play an important role in signal transduction in stresses and developmental processes. CBL-interacting Ser/Thr protein kinases have been demonstrated to function in different responses to abiotic stresses such as salt, drought, flood, wounding, abscisic acid (ABA), low and high temperature [68]. In our study, we observed that in tolerant species, TM-1 have two CBL-interacting Ser/Thr protein kinases. Previously, some researchers described that the maize (*ZmCIPK5*) might conduct calcium signals throughout *CBL/CIPK* complex under stresses and over-expression of *Gossypium hirsutum* (*GhCIPK6)* enhances tolerance to salt and drought as well as *ZmCIPK5* was up-regulated under PEG treatment [24,25]. Previous studies have shown that wheat (*TaCIPK14)* and *Arabiodopsis* (*AtCIPK14)* are up-regulated by treatments with salt, PEG, and ABA [68]. This result indicates that comparatively conserved mechanisms may be shared by cotton (TM-1) responses to various abiotic stresses such as drought.

Thaumatin-like proteins have been revealed to be involved in responses to salinity, cold, osmotic stress, and drought [69,70]. In *Arabidopsis*, several *YLS* (yellow leaf specific) genes were found to be up-regulated during normal senescence [71]. *YLS3*, a lipid transfer protein, was revealed to be up-regulated in the leaf yellowing, and this gene was up-regulated during the later stages of senescence [72]. In our recent study, we found that one *YLS3* and *TLPs* (*Thaumatin*) gene up-regulated in tolerant species (TM-1) and one down-regulated in sensitive species (Pima4-S) under drought stress conditions compared to their control. Rahman et al. [73] showed that the abundance of small heat shock proteins (sHSPs) were increased, ROS was decreased, and thereby photosystem II reaction is protected during drought stress. Some researchers also observed that sHSPs showed the strongest respond in maize and rapeseed under drought conditions [74]. In the present study, we observed that small heat shock protein, 18.1 kDa class I heat shock protein two proteins were up-regulated in tolerant species TM-1 under drought stress.

Various NAC TFs encoding genes were stress related, such as *ANAC019*, *ANAC055*, *ANAC072* [75], *ANAC029*, and *GmNAC072* [75]. Among *ANAC* TFs, *ANAC019, ANAC055*, and *ANAC072* with the functions as up-regulated in the drought stress, whereas *ANAC002* as a negative regulator in the drought stress response [75,76], and, moreover, *ERD1* was also expressed under dehydration and high-salinity stress conditions [77]. *ANAC019* and *ANAC055*, these two transcripts expressed to a higher level and play a major role in high salinity stress, and *ANAC072* mRNA, meanwhile, accumulated to high levels and play a vital role under dehydration in *Arabiodopsis*. The *NAC* TFs encoding gene is the most important role in the signal transduction pathways [75]. The *NAC* TFs genes were expressed under endogenous ABA and dehydration stress [78]. However, these *NAC* proteins *NAC072* may also regulate ABA biosynthesis-related genes such as *NCED3* and *RD22* might increase ABA biosynthesis due to their increased amount of transcripts in transgenic plants. The *NCED3* and *RD22* are important genes involved in drought stress conditions [12]. These genes are significantly up-regulated in drought-tolerant transgenic plants [79]. In our current study, the *RD22* gene was up-regulated only tolerant cotton species TM-1 [80] and *NCED3* gene was one up-regulated in TM-1 and one down-regulated in Pima4-S, and under drought stress condition the *NAC* domain protein 72 four was up-regulated in TM-1 and two up-regulated in Pima4-S cotton species under drought, compared with their control.

The *MYB44* improves stomatal closure to drought tolerance and plays a vital role in ABA signaling in *Arabiodopsis* [81]. In our current study, we found *MYB44* transcriptome factor which interacts with the ABA receptor proteins like *PYL*s, which bind to *PP2C*s, suggesting that *MYB44* is directly involved in ABA signaling in TM-1 cotton species [82,83]. Previously, we found that stomatal conductance was reduced in the tolerant species TM-1 less than the two other species (Zhongmian-16 and Pima4-S) [21], and recent studies identified that the transcriptomic factor *MYB44* was up-regulated in TM-1 under drought compare with control. Under stresses, different plants may favor one mechanism to specially scavenge ROS [84]. *TaODORANT1* were up-regulated in over-expressing tobacco under drought, and salt stress improved the stress-related genes. In our present study, we found that protein *ODORANT1* 3 up-regulated in TM-1 and 2 down-regulated in Pima4-S under drought stress. The result agreed with our previous studies that the stress tolerance of TM-1 was enhanced by up-regulating the expression of gene under drought stress conditions [85].

The *JUNGBRUNNEN1* (*JUB1*) is a regulator of drought stress in tomato and was revealed to improve drought tolerance. *JUB1* controls growth and increases tolerance to drought stress by consistent cellular pathways concerned with ROS and phytohormone biosynthesis/signaling [86]. *SlJUB1* expression is strongly persuaded under H_2_O_2_, NaCl, PEG, and dehydration treatments as well as regulation of abiotic stress response networks in tomato. The putative target genes of *SlJUB1* in tomato have two homologues, *SlDREB1* and *SlDREB2*. *DREB* genes have been reported to be functionally involved in the regulation of plant responses to drought stress, for example, soya bean *GmDREB2* [87], *GmERF3* [88], and tomato *JERF1* [89]. Authors reported that *SlJUB1* gene expression was decreased and increased the accumulation of H_2_O_2_, thus resulting in reduced tolerance to drought stress. In our previous study, we observed that in TM-1, the MDA level was low and photosynthetic rate was higher than two other cotton species [21]. Banana plants over-expressed *MusaNAC042*, the closest homologue of *AtJUB1*, which significantly reduced drought stress-induced oxidative damage by a lower level of MDA and a higher photosynthetic activity while improving drought and salinity stress [90]. In our present study, *JUNGBRUNNEN1* (*JUB1*) gene up-regulated only TM-1 tolerant cotton species.

Drought stress significantly declined leaf turgidity, cell expansion, canopy area, photosynthetic rate, and consequently reduces crop yield [91]. Various *AP2/ERF* transcription factors, such as *Bra-botrytis-AP2/ERF-2*, *Bra-botrytis-ERF106a*, *Bra-botrytis-ERF118a*, and *Bra-botrytis-RAP2-1*, increased their expression levels under drought stress [92]. In the current study, we observed that ethylene-responsive transcription factor *RAP2-1* was up-regulated in TM-1 species under drought stress condition. In Arabidopsis *RAP2.1*, the homolog of *Bra-botrytis-RAP2-1* was strongly induced by drought stress [93]. The role of *ICE1* has been extended in abiotic stresses, such as drought and salinity, and in some *ICE1* homologs such as *OsbHLH001*, *OsbHLH002*, and *CdICE1* [94]. Stomatal movements of leaves in response to the environment have been linked to drought tolerance and water use efficiency, which iswell documented [95]. Breaking of asymmetry in the stomatal lineage and speechless (SPCH) are necessary for producing a meristemoid. Furthermore, a guard mother cell can be separated from meristemoid by *MUTE* [96]. Guard cells were differentiated by *FAMA* [97]. In our study, we observed that *ICE1, MUTE, RAP2-1*, and *FAMA* transcriptional factors were up-regulated only tolerant species TM-1 under drought stress condition. Four transcriptional factors may be involved in drought tolerance activity in tolerant species TM-1. Therefore, it is possible to use these identified drought-responsive genes that are special to cotton. Further analysis of the functions and expression controlling mechanism of these genes in cotton would not only supply the opportunity of identification of novel genes, but also enlarge our further understanding of specific mechanismsof drought tolerance in cotton.

## 4. Materials and Methods

### 4.1. Plant Material and Growth Conditions

A pot experiment was conducted at the Zi-jin-gang campus, Zhejiang University, Hangzhou, China, to study comparative transcriptomic analysis of three cotton species on drought stress, namely, TM-1 (*Gossypiumhirsutum* L.), Zhongmian-16 (*Gossypiumarboreum* L.), and Pima4-S (*Gossypiumbarbadense* L.), in June 2016. The seeds of three cotton species were collected from the Institute of Cotton Research (ICR), Chinese Academy of Agricultural Sciences (CAAS) (Henan, China), and Zhejiang University, Hangzhou, China. The soil was collected from the experimental field of Zhejiang University, Hangzhou, China, and air-dried at 8% moisture content. The air-dried soil was put into plastic pots (7 L, 22 cm in height) and then fertilized with 1 L of a basal nutrient solution, as mentioned by Wu et al. [98]. The solution preparation was as follows (chemical name, µM): Ca (NO_3_)_2_·4H_2_O, 365.2; KNO_3_, 183.0; MgSO_4_·7H_2_O, 547.0; KH_2_PO_4_, 182.2; Fe-citrate, 19.5; K_2_SO_4_, 91.2; (NH_4_)2SO_4_, 365.2; MnCl_2_·4H_2_O, 4.5; ZnSO_4_·7H_2_O, 0.4; CuSO_4_·5H_2_O, 0.2; H_3_BO_3_, 46.9; H_2_MoO_4_, 0.1. The pH of the solution was adjusted to 5.6 ± 0.1 with NaOH or HCl as required. The pots were kept in a net house under natural light. The seeds of all three species were surface-sterilized in 3% H_2_O_2_ for 20 min and rinsed three times with distilled water. The seeds were sown in garden soil on germination trays in the tissue culture lab at a temperature of 30°C with 50% relative humidity (RH). Uniformly sized, 12-day-old seedlings were transplanted into 7-L plastic pots, three seedlings per pot, and were kept in the net house. Before the drought treatments, the seedlings were irrigated when necessary. The drought started when the plant had 5–6 leaves (30 DAT). The experiment was arranged in a completely randomized design (CRD) with four replications. Plants samples were collected from drought-applied pots when the soil moisture content was 4%. Soil moisture was measured using an HH2Moisture Meter (Delta-T Devices, Cambridge, UK). The young leaves (the second uppermost fully expanded) were immediately frozen in liquid nitrogen and then stored at –80°C for RNA extraction.

### 4.2. RNA Extraction and Illumina Sequencing

Three biological replicates were used for all RNA-Seq experiments from each control and drought stress. Total RNA was extracted from each sample using TRIzol Reagent (Invitrogen, Carlsbad, CA, USA)/RNeasy Mini Kit (Qiagen, Hulsterweg, PL, The Netherlands). Total RNA was quantified and qualified by Agilent 2100 Bioanalyzer (Agilent Technologies, Palo Alto, CA, USA), NanoDrop (Thermo Fisher Scientific Inc., Pforzheim, Germany), and 1% agrose gel. Then, 1 μg total RNA with a RIN value above 7 was used for the following library preparation. Next generation sequencing (NGS) library preparations were constructed according to the manufacturer’s protocol (NEBNext^®^ Ultra™ RNA Library Prep Kit for Illumina^®^). The poly mRNA isolation was performed using NEBNext Poly(A) mRNA Magnetic Isolation Module (NEB) or Ribo-Zero™ rRNA removal Kit (Illumine, San Diego, CA, USA). The mRNA fragmentation and priming were performed using NEBNext first Strand Synthesis Reaction Buffer and NEBNext Random Primers. First strand cDNA was synthesized using ProtoScript II Reverse Transcriptase and the second-strand cDNA was synthesized using Second Strand Synthesis Enzyme Mix. The purified double-stranded cDNAbyAxyPrep Mag PCR Clean-up (Axygen) was treated with End Prep Enzyme Mix to repair both ends and add a dA-tailing in one reaction, followed by a T-A ligation to add adaptors to both ends. Size selection of Adaptor-ligated DNA was performed using AxyPrep Mag PCR clean-up (Axygen), and fragments of ~360 bp (with the approximate insert size of 300 bp) were recovered. Each sample was amplified by PCR for 11 cycles using P5 and P7 primers, with both primers carrying sequences which can anneal with flow cell to perform bridge PCR and P7 primer carrying a six-base index allowing for multiplexing. The PCR products were cleaned up using AxyPrep Mag PCR Clean-up (Axygen). They were validated using an Agilent 2100 Bioanalyzer (Agilent Technologies, Palo Alto, CA, USA), and quantified by Qubit 2.0 Fluorometer (Invitrogen, Carlsbad, CA, USA). Libraries with different indices were multiplexed and loaded on an Illumina HiSeq instrument according to manufacturer’s instructions (Illumina, San Diego, CA, USA). Sequencing was carried out using a 2 × 150 bp paired-end (PE) configuration; image analysis and base calling were conducted by the HiSeq Control Software (HCS) + OLB + GAPipeline-1.6 (Illumina).

### 4.3. Differential Gene Expression (DGE) Analysis

Reference genome sequences and gene model annotation files of cotton species were downloadedfrom a genome website, such as UCSC, NCBI, ENSEMBL, and Hisat2 (v2.0.1) was used to indexreference genome sequence. Clean data were aligned to reference genome via software Hisat2 (v2.0.1) [99]. In the beginning, transcripts in FASTA format wereconverted from known GFF annotation file and indexedproperly. Then, with the file as a reference gene file, HTSeq (v0.6.1) estimated gene and isoformexpression levels from the pair-end clean data.Differential expression analysis used the DESeq Bioconductor package, a model based on the negativebinomial distribution. After being adjusted by Benjamini and Hochberg’s approach for controlling the falsediscovery rate, the *p*-value of genes were set <0.05 to detect differential expressed ones, acquisition of clean reads, and mapping.

### 4.4. GO and KEGG Pathway Analysis

Functional enrichment analysis including GO and KEGG was performed to identify which DEGs were significantly enriched in GO terms or metabolic pathways. DEGs are mapped to the GO terms (biological functions) in the database, the number of genes in every term is calculated, and a hypergeometric test is performed to identify significantly enriched GO terms in the gene list out of the background of the reference gene list. The Kyoto Encyclopedia of Genes and Genomes (KEGG) database is public pathways or signal transduction pathways enriched in DEGs compared to a reference gene background, using the hypergeometric test. GO terms and KEGG pathway with false discovery rate (*q*-value) < 0.05 were considered as significantly altered. Based on DEGs and a 10% tree cutoff, hierarchical clustering was performed using the R package cut tree.

### 4.5. Validation of DEGs by RT-qPCR

For RNA-seq data validation, we choose 13 genes randomly from DEGs and tested their expression level at early heading stages using qRT-PCR, which were the exact same RNA samples that wereused for RNA-seq. The cDNA was synthesized from isolated RNA by reverse transcriptase using the cDNA synthesis kit (GoScript™ Reverse Transcription System, Promega, Beijing, China). RT-PCR was performed using the SYBR Green Master Mix (Applied Biosystems™ SYBR™ Green RT-PCR Master Mix, Waltham, MA, USA) with an ABI 7500 Real-Time PCR system (Applied Biosystems^®^ 7500 Real-Time PCR Systems, Waltham, MA, USA). The total reaction volume was 15 µL, containing 1 µL of cDNA, 7.5 µL of 2× SYBR Green Master Mix (Applied Biosystem, Waltham, MA, USA), 1 µL of primer mix (0.5 µM), 0.3 µM of Rox, and 5.2 µL of ddH2O. All samples were amplified in triplicate assays using the following conditions: 95 °C for 2 min for 1 cycle followed by 40 cycles at 95 °C for 15 s, 55 °C for 30 s, and 72 °C for 30 s, and a final extension at 72 °C. The gene-specific primers were designed using PRIMER3 (https://www.ncbi.nlm.nih.gov/tools/primer-blast/). The primer details are listed in Table S3. The cotton EF1α (EF1α-F: 5-AGACCACCAAGTACTACTGCAC-3; EF1α-R: 5 CCACCAATCTTGTACACATCC-3) gene was used as an endogenous control for all the qRT-PCR analyses. The relative transcription levels were calculated using the 2^−∆∆*C*t^ method [100].

## 5. Conclusions

In conclusion, the transcriptome data was used to identify and interpret a large number of DEGs involved in drought stress in cotton species, which provides an admirable platform for future genetic and functional genomics analysis. Genes related to drought tolerance and their expression profiles at the vegetative stage were analyzed further. This offered insights into the molecular mechanisms underlying cotton drought tolerance. In addition, the current results provide some candidate genes, which can be used in cotton breeding for improving drought tolerance. The up-regulated DEGs included some previously reported genes (e.g., NAC, FAMA, MUTE, bZIP, ICE1, *JUNGBRUNNEN1*, and *MYB*) and some new genes (e.g., *PYL, PP2C, SRK2E, Thaumatin, ODORANT1, NCED3*, and *RD22*) that are thought to be related to drought tolerance. We also identified in GO analysis biological process category present only in the TM-1 and KEGG pathway, a hormonal signal transduction pathway involved in drought tolerance in TM-1 species. The data presented here gives an insight into the molecular changes underlying the drought tolerant in TM-1 (*Gossypium hirsutum*), and the results can be used as a reference for identifying candidate genes that hold great potential for the creation of novel germplasms with enhanced drought stress tolerance.

## Figures and Tables

**Table 1:** Descriptive statistical analysis of RNA-seq.

Summary	Min	Max	Total	Average
Total Raw reads	49,311,644	64,712,390	326,938,772	54,489,795.3
Total clean reads	48,199,244	63,896,272	322,114,206	53,685,701
Total clean reads/total Raw reads	98%	99%	99%	99%
Total clean bases	7,111,623,297	9,450,136,133	47,633,258,301	7,938,876,384
GC content	41.88%	43.60%	--	42.99%
Q20	96.26%	96.76%	--	96.49%
Q30	91.18%	92.17%	--	91.59%
Total mapped reads	38,646,418	50,124,573	262,172,868	43,695,478
Total mapped reads	78.45%	84.66%		82%
Multiple mapped	4751,886	6,451,220	34,568,807	5,761,467.83
Multiple mapped%	9.67%	12.10%	--	10.75%
Uniquely mapped	33,372,748	43,788,024	227,604,061	37,934,010.2
Uniquely mapped%	68.49%	74.98%	--	71%
Total mapped reads/Total clean reads	80%	78%	81%	81%

## Supplementary Materials

Supplementary materials can be found at https://www.mdpi.com/1422-0067/20/9/2076/s1. Figure S1. RPKM plot box distribution of three cotton species. Figure S2. RPKM density distribution in three cotton species. Figure S3. Distribution of reads in different regions of three cotton genome. Figure S4. Percent error rate saturation curve. Figure S5. Venn diagram is showing that total of 6968 DEGs. Figure S6. Differential expression genes MA plot and volcano plot. Figure S7. Cluster analysis of DEGs under GO terms is depicted in the Heat map. Table S1–S6: Zip file of all related data set and name list.
