# Supplementary material for: Comparative Transcriptomic Analysis of Biological Process and Key Pathway in Three Cotton (Gossypium spp.) Species Under Drought Stress
Source: Int J Mol Sci. 2019 Apr 27;20(9):2076. doi: 10.3390/ijms20092076 (PMC6539811; doi:10.3390/ijms20092076)
Supplement: Supplementary file 1 [file ijms-20-02076-s001.pdf]

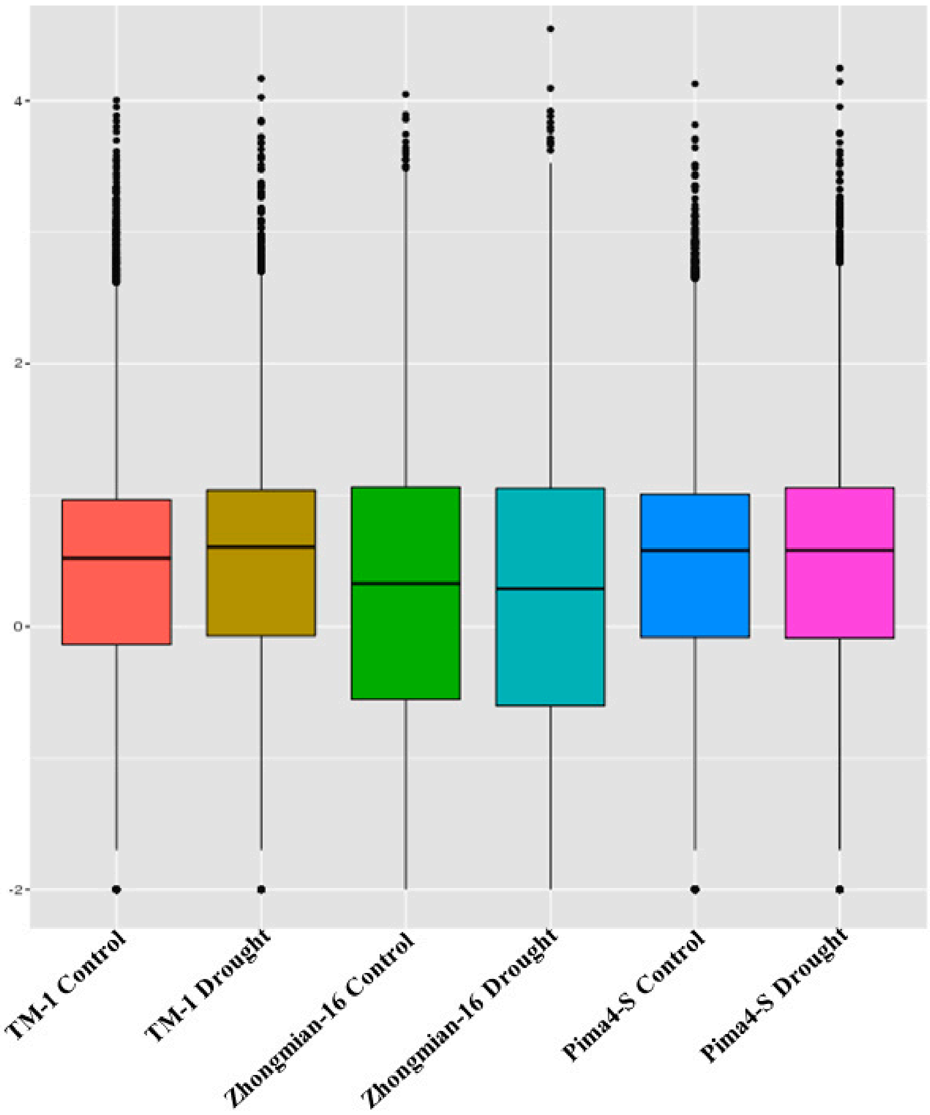

Figure S1. Reads Per Kilobase Million (RPKM) plot box distribution of three cotton species.

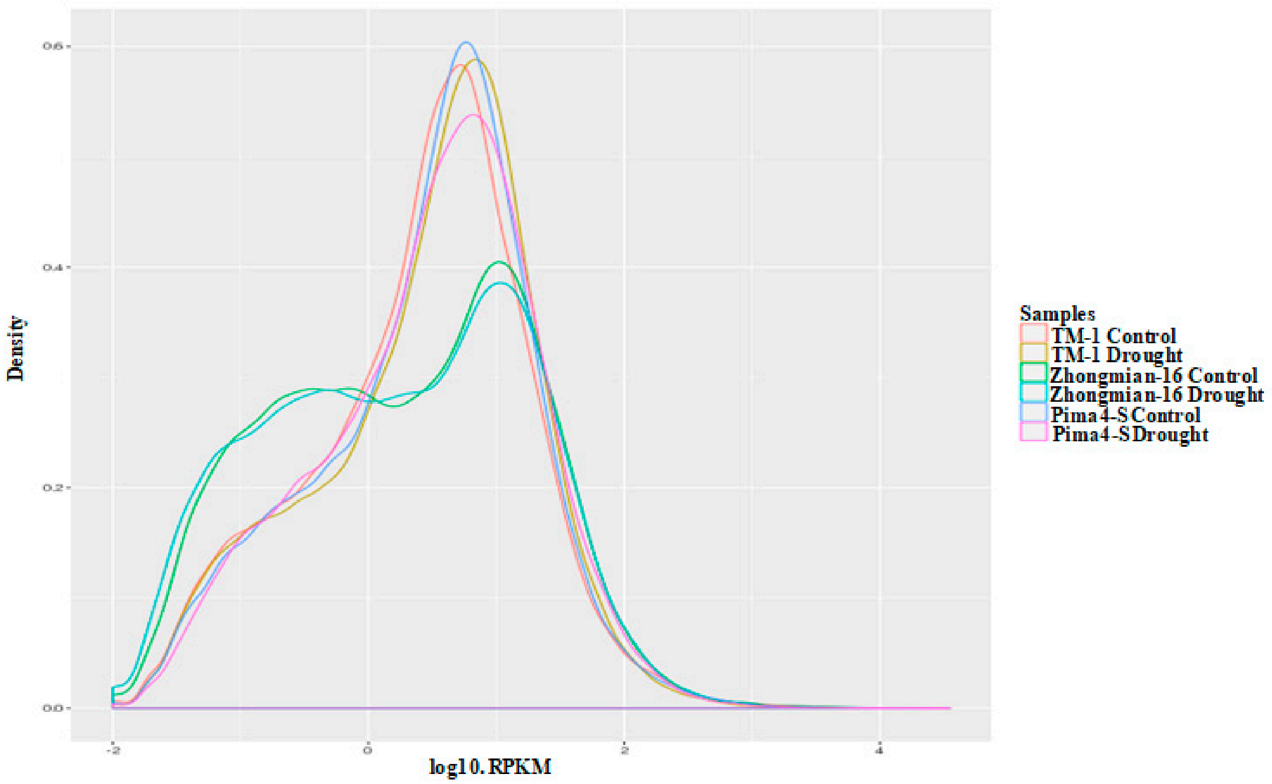

Figure S2. RPKM density distribution in three cotton species.

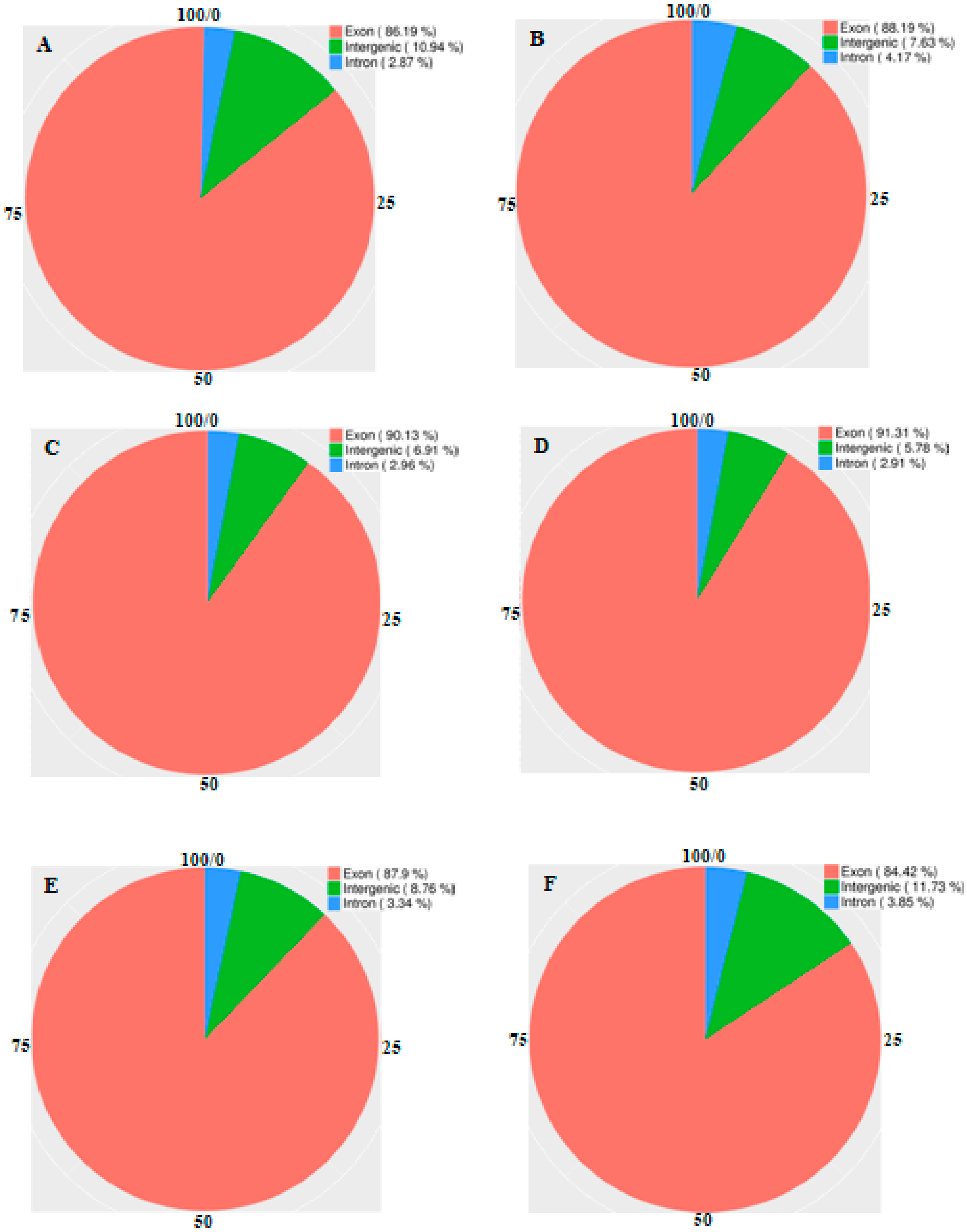

Figure S3. Distribution of reads in different regions of three cotton genome. Here, A) TM-1 Control, B) TM-1 Drought, C) Zhongmian-16 Control, D) Zhongmian-16 Drought, E) Pima4-S Control, and F) Pima4-S Drought.

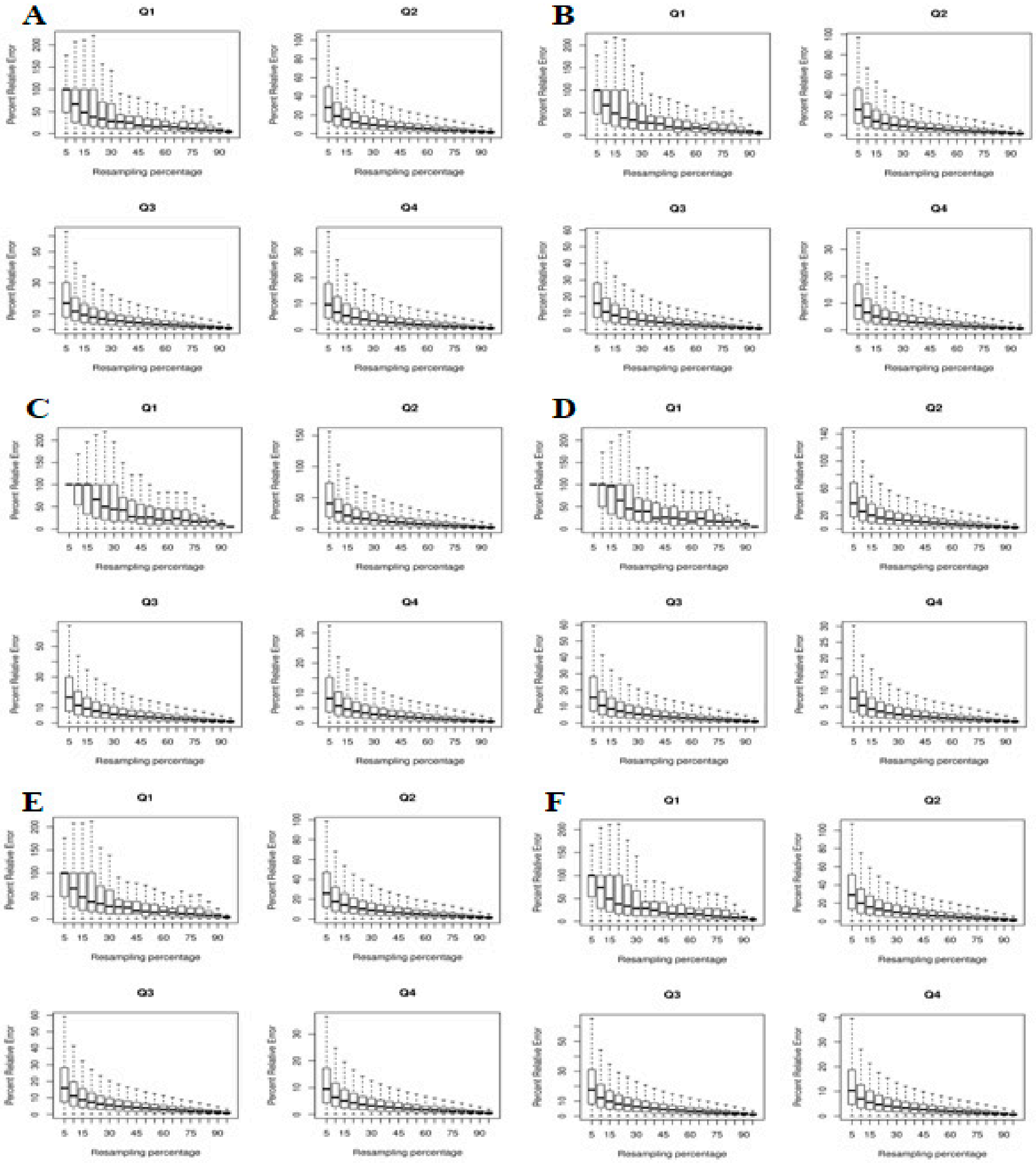

Figure S4. Percent error rate saturation curve. X axis: percentage of sampling reads. Y axis: Percent relative error. Saturation curve A) TM-1 Control, B) TM-1 Drought, C) Zhongmian-16 Control, D) Zhonmian-16 Drought, E) Pima4-S Control, and F) Pima4-Sdrought (Q1 is a saturation box plot with transcript expression levels below 25%; Q2 is a saturation box plot with transcript expression levels between 25% and 50%; Q3 is a saturation box plot with transcript expression levels between 50%, and 75%; Q4 is the saturation box plot of transcript expression levels above 75%).

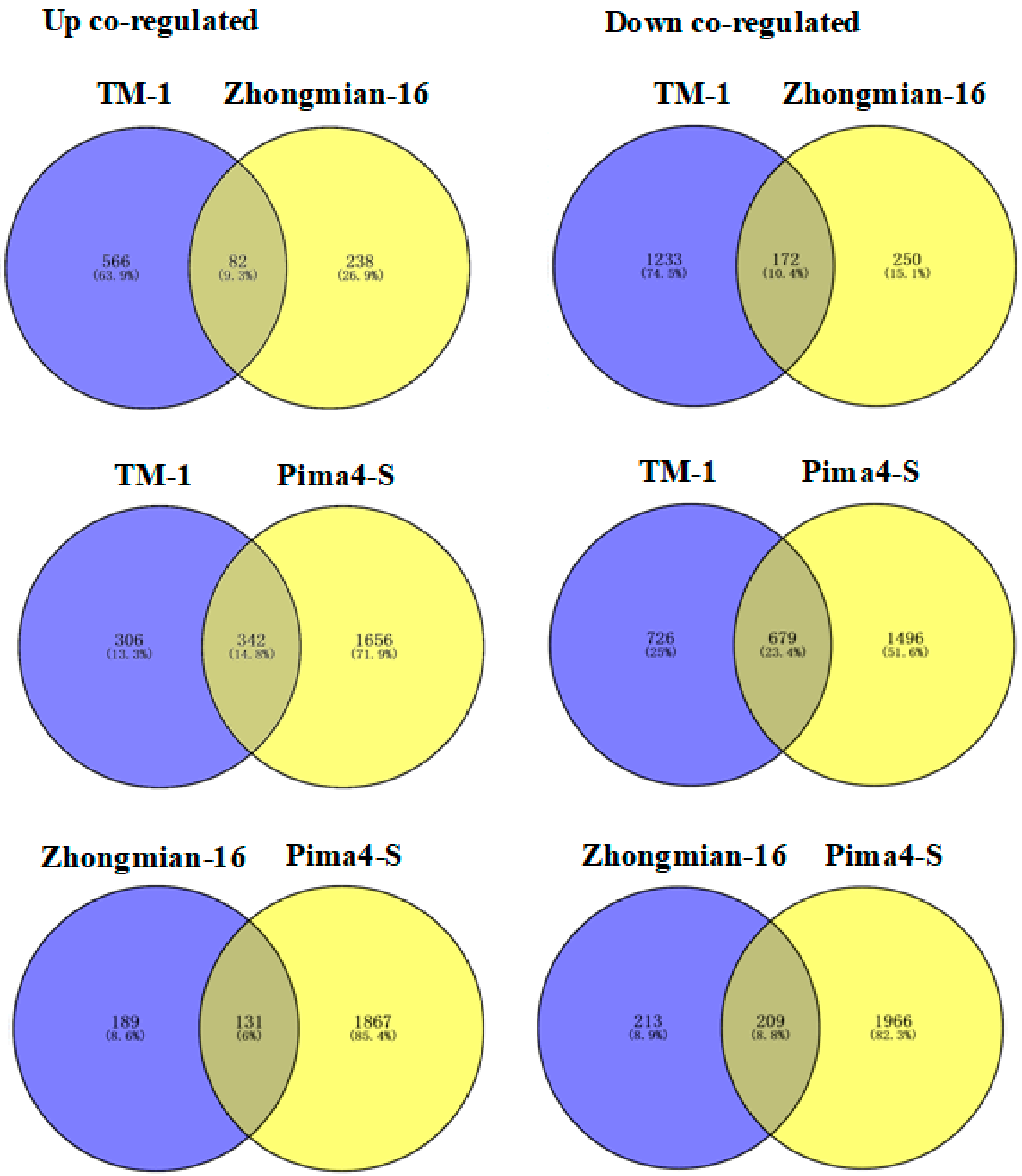

Figure S5. Venn diagram showing the total of 6968 DEGs. The number of up co-regulated DEGs in TM-1, Zhongmian-16, and Pima4-S (Control-vs-Drought) and down co-regulated DEGs in TM-1, Zhongmian-16, and Pima4-S (Control-vs-Drought) were found in each comparison.

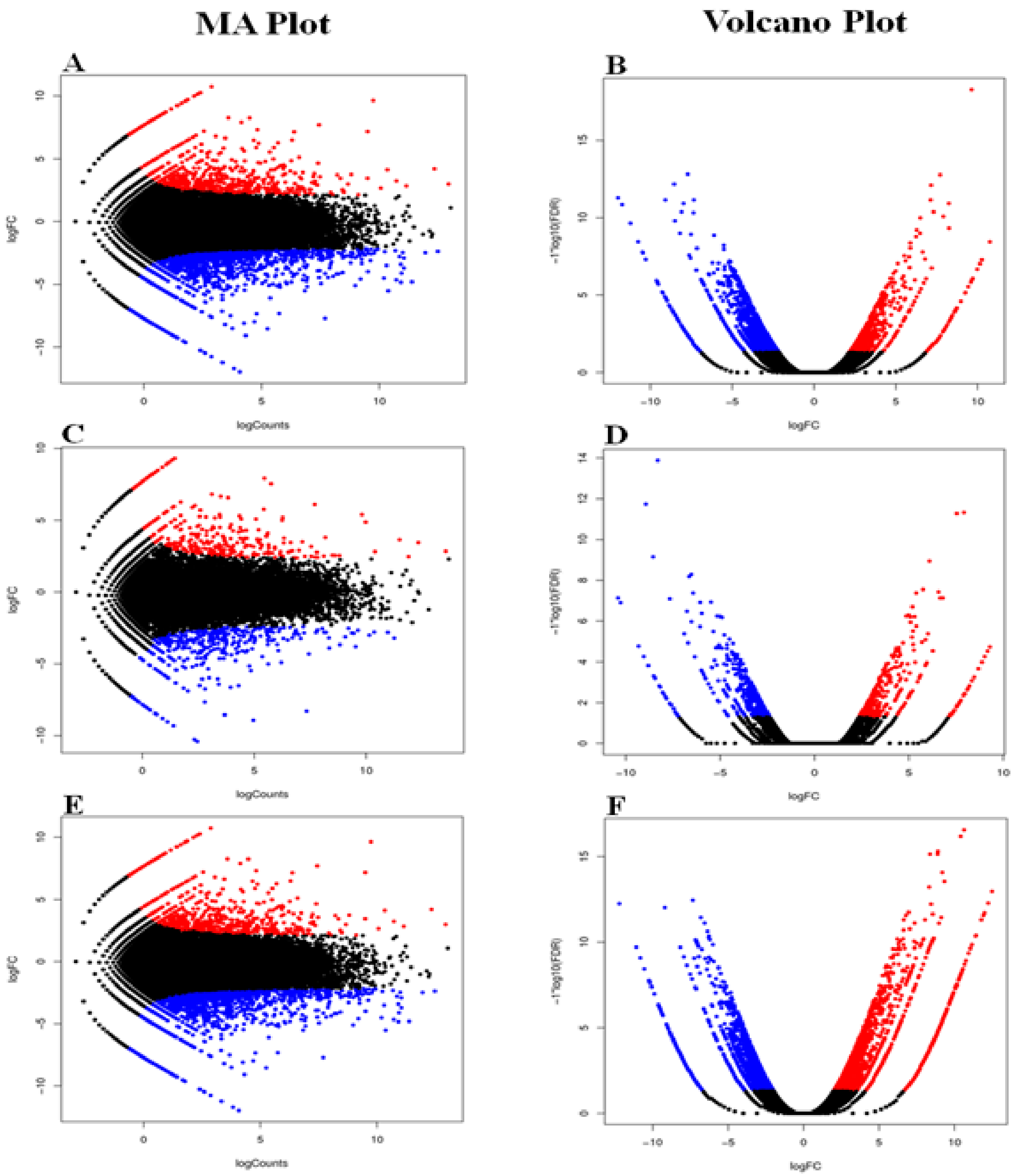

Figure S6. Differential expression genes MA plot and volcano plot. Here, red dots represent genes that are significantly up-regulated and blue dots represent down-regulated genes. A,B) TM-1 Control-vs-Drought, C,D) Zhongmian-16 Control-vs-Drought, and E,F) Pima4-S Control-vs-Drought. X axis: log2 fold change of gene expression. Y axis: statistical significance of the differential expression in log10 (FDR).

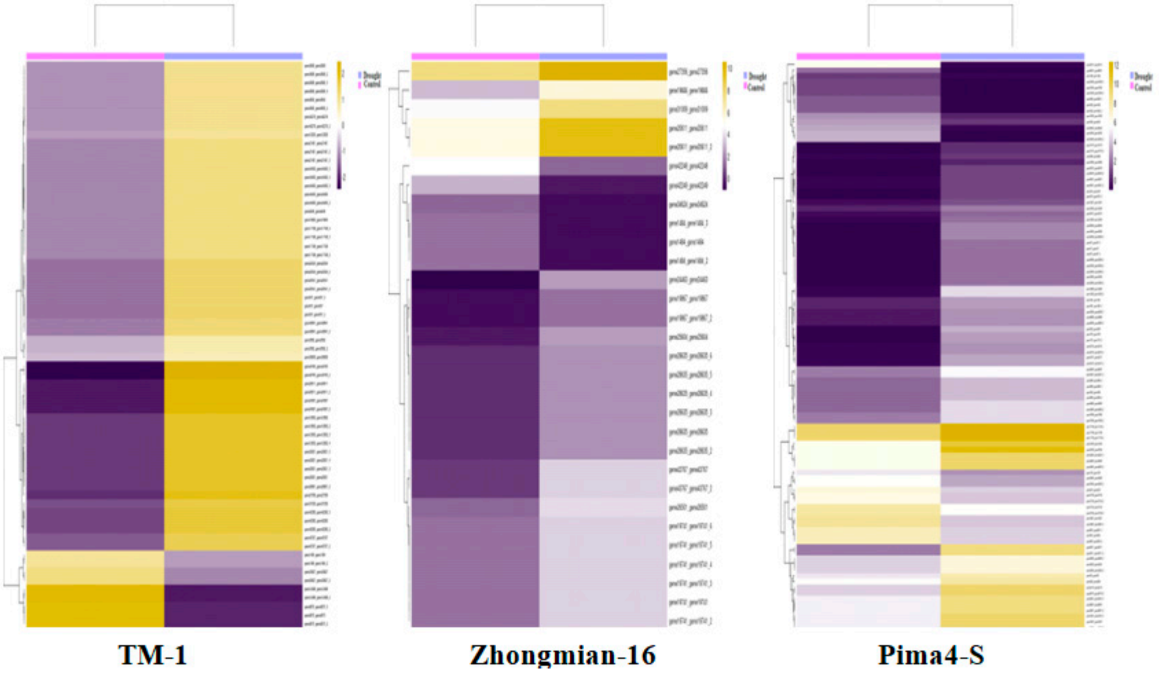

Figure S7. Cluster analysis of DEGs under GO terms is depicted in the Heat map. Here, TM-1 control and drought, Zhongmian-16 control and drought, Pima4-S control and drought; Yellow color indicates up-regulated genes and magenta indicates down-regulated genes.
